# Improving Medical Students’ Confidence in Performing Mental State Examinations: A Quality Improvement Project Using Creative and Narrative Teaching Methods, Video-Based Learning, and Documentation Practice

**DOI:** 10.1192/bjo.2025.10305

**Published:** 2025-06-20

**Authors:** Clara Seipt, James Anderson

**Affiliations:** DPT, Exeter, United Kingdom

## Abstract

**Aims:** Starting a new role as Clinical Teaching Fellows, early student feedback identified a gap between students’ academic understanding of the Mental State Examination (MSE), and confidence in its application and interpretation. Assessors recognised similar uncertainty in students regarding findings in the MSE, and a lack of confidence in presenting. This project aimed to improve medical students’ self-reported confidence in the MSE via an interactive workshop. Each PDSA cycle, we aimed to implement feedback suggestions through creative teaching methods, to improve confidence, engagement, and interaction in the MSE teaching.

**Methods:** Year 3 and 4 medical students attended the MSE workshop during their rotation in Psychiatry. Quantitative and qualitative feedback was gathered via feedback surveys, accessible via a QR code. Using a Likert scale, students rated their confidence performing the MSE before and after the workshop. Thematic analysis of the qualitative feedback was undertaken to explore attitudes, aspects most enjoyed, and suggestions for improvement. The workshop began with simulated videos to explore and develop knowledge on the MSE. In the second PDSA cycle, we added a creative small group task, asking students to perform the MSE on a fictional/famous character and present back to the group. Finally, a documentation task was added whilst students observed a simulated patient interview.

**Results:** 64 students participated in the MSE Workshop feedback survey. Students reported an average confidence rating of 56.0% prior to the session. After the workshop, the average confidence level increased to 86.8%. Furthermore, 76.6% of students rated the session as “extremely useful” for improving their skills in MSE when compared with previous teaching at medical school. 81.3% would “definitely” recommend the workshop to other medical students.

Qualitative data showed the use of narrative videos was well received by students, with 16 responses highlighting this as a strength of the workshop. “I loved the example videos; helping to clarify and talking through it afterwards was exceedingly helpful!” The opportunity to practice documenting the MSE was another theme within the positive feedback (7 responses). Students highlighted the interactive elements, clarity, and structure as further strengths. Suggestions to improve the session included activities to support phrasing of questions to patients and promoting consolidation using a quiz.

**Conclusion:** The workshop increased students’ confidence in MSE performance. Students appreciated the use of creative elements, video examples, and documentation tasks. Future improvements could be made to support communication skills, question phrasing, and promote engagement via game-based knowledge assessments.

